# Barriers and motivations: analyzing the factors influencing abortion provision by gynecologists in Germany – a cross-sectional study

**DOI:** 10.1186/s12978-026-02389-8

**Published:** 2026-06-18

**Authors:** Vanessa Le, Eva Christalle, Mareike Rutenkröger, Jördis Zill, Isabelle Scholl, Anja Lindig

**Affiliations:** 1 Department of Medical Psychology, University Medical Center Hamburg-Eppendorf, Martinistraße 52, Hamburg, 20246 Germany; 2 Department of Psychology, Faculty of Medicine and Health Sciences, University Medicine Oldenburg, Carl von Ossietzky University Oldenburg, Ammerländer Heerstraße 138, Oldenburg, 26129 Germany

**Keywords:** Abortion, Gynecologists, Abortion providers, Abortion care, Stigma, Theory of planned behavior

## Abstract

**Background:**

The influence of factors like attitudes, subjective norms, or perceived behavioral control on physicians’ intention to provide abortion care in Germany is not evaluated so far. We applied the Theory of Planned Behavior to analyzes the role of those factors, and compare providers and non-providers to identify possible barriers to abortion provision amongst physicians.

**Methods:**

A cross-sectional online survey was conducted between June and November 2024 among office-based gynecologists and abortion providers of all specialties, recruited via cluster sampling. The survey assessed a range of factors influencing the provision of abortion, including attitudes, subjective norms, perceived behavioral control, religiosity, fear of stigmatization, gender and knowledge. Multiple regression analysis tested the TPB model, abortion providers and non-providers were compared via t-tests.

**Results:**

*N* = 213 participants completed the survey. Perceived behavioral control (β = 0.479, *p* < 0.001) explained the most variance in behavioral intention to provide abortions, followed by subjective norms (β = 0.326, *p* < 0.001) and attitudes (β = 0.136, *p* = 0.010). The group comparisons between abortion providers and non-providers revealed that abortion providers showed more positive attitudes towards abortions, stronger perceived subjective norms on providing abortions, higher perceived behavioral control of providing abortions, lower fear of stigmatization, lower religiosity and higher subjective knowledge on abortions and legal regulations than non-providers.

**Conclusions:**

Intentions to provide abortion care are influenced by attitudes, subjective norms, and especially perceived behavioral control, indicating a need for targeted interventions addressing those factors to improve intentions to provide abortion care.

**Supplementary Information:**

The online version contains supplementary material available at 10.1186/s12978-026-02389-8.

## Abstract (plain language summary)

This study aimed to understand why some doctors are more likely to offer abortion services than others. To do this, we used a psychological model called the *Theory of Planned Behavior*, which looks at how a person’s attitudes, social influences, and sense of control affect their intentions and actions. We surveyed 213 doctors between June and November 2024, including gynecologists and other specialists. The survey asked about their attitudes towards abortion, how much pressure or support they feel from others, how confident they feel in providing abortions, their religious beliefs, fear of stigma, and how much they know about abortion laws. We found that the strongest factor influencing a doctor’s intention to provide abortions was their confidence in being able to do so. Social support and personal attitudes also played important roles. Doctors who do not provide abortions differ from those who provide abortions. Doctors who provide abortions are more confident, feel more supported by others, have more positive attitudes, fear stigma less, are generally less religious, and know more about abortion and related laws. These results can be used to develop strategies for doctors to increase their intention to provide abortions.

**Supplementary Information:**

The online version contains supplementary material available at 10.1186/s12978-026-02389-8.

## Background

In Germany, abortions are regulated under §218 and §219 of the German Criminal Code (German: “Strafgesetzbuch”, StGB) [1] and are classified as unlawful. However, they remain exempt from punishment if performed by a physician within the first twelve weeks of pregnancy post conceptionem (p.c.), i.e., after conception/fertilization, following mandatory state-recognized pregnancy conflict counselling and a three-day waiting period (so called “counselling regulation”). It is also exempt in cases of medical or criminological indication. Individual physicians and institutions have the right to conscientious objection and may refuse to perform an abortion due to ethical or religious reasons [1]. In 2024, 106,455 abortions were recorded in Germany (63 abortions per 10,000 women) compared to 677,117 live births [2, 3]. Approximately 96% of all abortions in Germany are performed under the counselling regulation, mostly in outpatient settings by gynecologists [4]. Methods recommended by the World Health Organization (WHO) and national guidelines for up to 14 weeks of pregnancy post menstruationem (p.m.), i.e., after the onset of menstruation, are medical abortion (drug combination of mifepristone and misoprostol, alternatively misoprostol alone), and surgical abortion via vacuum aspiration [5, 6]. There is high regional differences in availability and accessibility of abortion providers in Germany and therefore limited access to abortion services in several German regions [2, 7].

Against the backdrop of legally regulated yet uneven regional provision of abortion services, it is crucial to grasp the barriers that hinder access to care. We already knew several barriers for persons seeking abortions in Germany, including stigmatization, costs and organizational barriers, such as longer waiting times and longer travel distances [8–11]. However, while barriers on the patient side have been increasingly documented, we know less about attitudes of German abortion-providers and non-providers towards abortion, nor about other influencing factors on their intention to provide abortions. Identifying those factors and analyzing differences between providers and non-providers could support the development of strategies to sustain abortion services. To better understand such decision-making processes among physicians, theoretical frameworks from behavioral science can be applied. International research shows that influencing factors on physicians’ behavior can be described by Ajzen’s Theory of Planned Behavior (TPB) [12–14]. According to this theory, it can be assumed that three aspects influence the *behavioral intention* of physicians to provide abortions: (1) *attitudes* towards abortions, (2) *subjective norms* of providing abortions and (3) *perceived behavioral control* of providing abortions. Furthermore, the *behavioral intention* directly influences the actual *behavior*.

In addition to these theoretical constructs, previous research has identified a number of individual and contextual factors that have shown to influence abortion provision – and thus may also influence physicians’ intentions regarding the provision of abortions. International and national studies have identified *fear of stigmatization* [15–17], *religiosity* [15, 18, 19], *gender* [20], *subjective knowledge on abortions* [15, 19, 21] and *subjective knowledge on current legal regulations of abortions* [22] as influencing factors on abortion provision. Both international and national studies have described the role of stigmatization in abortion providers’ past experiences [23, 24] as well as their fear of stigmatization in the context of providing abortions [11, 16, 17]. A qualitative German study from Baier et al. (2023) identified *fear of stigmatization* as a barrier for abortion provision regardless of whether abortions are offered by the interviewed (future) gynecologists or not [15]. Strong *religiosity* has shown to be associated with not offering abortion due to religious beliefs [15, 18, 19, 25]. Furthermore, former studies also showed that female physicians are more likely to provide abortions [20, 25]. Theoretical and practical *knowledge about abortion* [15, 19, 21] and *knowledge on current legal regulations* [22] also play an important role in determining whether to provide abortions or not. Baier et al. (2023) described that misconceptions on abortion reduce physicians’ willingness to perform abortion in Germany [15]. Lack of training, and therefore lack of knowledge, has shown to be an important factor for a reduced intention of medical students to provide abortion care [19].

Despite these insights, there is still limited empirical evidence for the German context. To date, there is no current nationwide study in Germany that examines the relationships between the above-mentioned influencing factors and intention to abortion provision. Given the distinct legal regulations on abortion in Germany, the characteristics of the German health care system, and cultural contexts, results of international studies cannot be transferred to the German context. Therefore, the study at hand aims to analyze the influence of *attitudes* towards abortion, *subjective norms* of providing abortions and *perceived behavioral control* of providing abortion on the *behavioral intention* to provide abortions based on the TPB. Furthermore, the influence of *gender*,* religiosity*,* fear of stigmatization and subjective knowledge on behavioral intention to provide abortions* should be assessed *and* abortion *providers* and *non-providers* should be compared regarding the characteristics of all factors mentioned.

## Methods

### Study design

This quantitative cross-sectional study was conducted to analyze the influence of several individual factors on the intention to provide abortions. Within this study we aimed to compare physicians offering abortions (*providers’*) with those not offering abortions (*non-providers’*). Thereby, office-based gynecologists (regardless of their contribution to abortion care) and abortion providers, which include physicians of other specialties (e.g. general medicine), in Germany were included. Exclusion criteria for the *‘non-provider’* group were employment in the outpatient sector and working in a hospital, as their individual (intention to) abortion provision may depend on directives of their employer, whether to offer abortions or not. Thereby, abortion *providers* could include office-based and employed outpatient gynecologists as well as physicians of other specialties. *Non-providers* only included office-based gynecologists.

This study was conducted linked to the project “Assessment of person-centeredness in healthcare and social support services for women with unintended pregnancy (CarePreg)” [26], which was funded by the German Federal Ministry of Health (German: Bundesministerium für Gesundheit, BMG) and which evaluated person-centeredness within medical and psycho-social care for unintended pregnant women seeking abortions in Germany. The study at hand was not funded by the BMG.

We developed an online survey assessing 15 constructs via 93 items in total (for an overview of constructs see Table 1). The survey was finalized after feedback from nine physicians providing abortions, who were collaborating partners in the CarePreg study. As a result, a few items have been adapted, added or deleted. The survey was entered into LimeSurvey, and members of our research group tested the survey for errors, such as spelling mistakes, for comprehensibility and completion time.

**Table 1:** Assessed constructs and example items

Construct	Number of items	Example item	Source
Demographic data	8	How old are you?	Self-developed
Religiosity	2	How strongly do your religious beliefs influence your thoughts and actions?	Adapted [18]
Behavior	9	Do I currently offer medical abortions?	Self-developed
Reasons for non-provision^a^	6	Why do you think many gynecologists do not offer abortions?	Self-developed
Questions about the abolition of §219a^a^	4	Did you provide information on the topic of abortion on your website after the abolition of §219a?	Self-developed
Attitudes (AAS)	20	Abortions are wrong under all circumstances.	Validated [27]
Subjective norm	3	I am expected to offer abortions.	Adapted [28]
Perceived behavioral controls	5	The decision to offer abortions is in my hands.	Adapted [28]
Behavioral intention	3	I would like to offer abortions in the future.	Adapted [28]
Fear of stigmatization (APSS)	10	The reaction of other people to the fact that I perform abortions would make me keep a low profile.	Validated [29]
Fear of criminalization^a^	1	I would be afraid of criminalization through the current legal regulation of abortion.	Self-developed
Subjective knowledge on abortion	6	I consider myself well informed about the state laws and regulations on abortion.	Self-developed
Contact during training^a^	3	Would you have liked to have learned more about performing abortions during your specialist training?	Self-developed
Perception on the current care situation^a^	4	Unintended pregnant women have easy access to abortions in Germany.	Self-developed
Objective Knowledge on abortion^a^	9	The costs for abortion (according to counselling regulations) are generally covered by health insurance.	Adapted [30] and self-developed

*AAS*  Abortion Attitude Scale, *APSS*  Abortion Provider Stigma Scale

^a^Items were not included in data analysis, but descriptive statistics can be found in Additional file 2

A priori sample size calculation was conducted using GPower 3.1, for the multiple regression analysis and t-test. With a confidence level of 95%, an alpha error of 0.05, a planned power of 0.95 and an expected moderate effect size, the required number of cases was *n* = 74 for multiple regression analysis with three predictors and *n* = 176 for the t-tests for two independent groups.

The Checklist for Reporting Results of Internet E-Surveys (CHERRIES) [31] was filled out and can be found in Additional file 1. This study was preregistered on the Open Science Framework (OSF) platform before data collection (Identifier: osf-registrations-jh7a8-v1).

### Measures

Relevant constructs, scales and items were chosen based on a literature review on barriers to abortion care and factors influencing the (intention to) provide abortions. Single items were added after feedback by collaborating physicians providing abortion care. Constructs assessed in the online survey included *behavior*,* reasons for non-provision*,* questions about the abolition of § 219a*,* the variables of the TPB (attitudes*,* subjective norms*,* perceived behavioral control*,* reasons for non-provision intention*,* behavior)*,* fear of stigmatization* and *criminalization*,* subjective and objective knowledge about abortion* and *the current legal regulation of abortion*,* the perception of the current care situation*,* education experiences regarding abortion care* (e.g. within medical school or residency) as well as *demographic data* (including questions on *religiosity* and *gender*).

Items of the constructs *demographic data*,* religiosity*,* behavior*,* attitudes*,* subjective norms*,* perceived behavioral control*,* behavioral intention*,* fear of stigmatization and subjective knowledge on abortions* (including *subjective knowledge on current legal regulations of abortions*) were mandatory, and the results are presented in this paper. Further voluntary items assessing the constructs *attitudes*,* reasons for non-provision*,* questions about the abolition of § 219a*,* fear of criminalization*,* questions on availability of abortion care*,* questions about education* and *objective knowledge on abortion* were deliberately excluded from the data analysis due to non-validation and were only described descriptively in Additional File 2. Validated scales such as the Abortion Attitude Scale (AAS) [27] and the subscale ‘Disclosure management’ of the Abortion Provider Stigma Scale (APSS) [16] were used. We selected only one subscale of the APSS to avoid overlength of the questionnaire. This subscale of the APSS was suitable for the survey, as its content also fits to the perspective of non-providers. The items on variables of the TPB were created using a manual for constructing questionnaires based on the TPB by Francis et al. [28]. Several items were adapted from similar studies [18, 30] or were self-developed. For an overview of constructs and example items, see Table 1. Details on analyzed constructs and measures can be found in Additional file 3. The AAS, the APSS and the items for the TPB were translated from English to German by applying the TRAPD (= Translation, Review, Adjudication, Pretest and Documentation) protocol [32].

The survey started with a study description containing information on the aim of the study and data protection, questions on the *inclusion criteria* (working as an office-based gynecologist and/or providing abortions), followed by the above-mentioned scales and items. Participants were asked to refer their answers to abortions conducted by counselling regulation (before the 12th week of pregnancy p.c.) unless otherwise requested. The whole survey can be found in Additional file 4 in its original language.

### Data collection

Data were collected from June to November 2024. We used a cluster sampling method and selected one large city, two medium-sized towns, three small towns and five rural communities from 13 federal states in Germany (*n* = 146 in total). Therefore, we used ChatGPT-4 with prompts like “Name 2 random medium-sized towns (between 20,000 and 100,000 inhabitants) in Bavaria”. The three German city-states (Berlin, Bremen, Hamburg) were added to the category of large cities. E-mail addresses from all outpatient gynecologists within the selected cities, towns and communities were manually collected via an official national health website [33]. In the first round, *n* = 963 e-mails were sent to the selected gynecologists and gynecological practices containing the study invitation and a link to the online survey. These e-mails were followed by three reminder e-mails. In addition to the cluster sampling, *n* = 226 abortion providers (based on a list of the German Medical Association provided by Doctors For Choice Germany) were contacted via e-mail. These email addresses have been removed previously from the cluster sampling mailing list to avoid duplication. Study invitations and the survey link were also distributed via networks of the following gynecological societies: Working Group of Women’s Health in Medicine, Psychotherapy and Society (German: Arbeitskreis Frauengesundheit in Medizin, Psychotherapie und Gesellschaft e.V., AKF), the German Society for Psychosomatic Gynecology and Obstetrics (German: Deutsche Gesellschaft für Psychosomatische Frauenheilkunde und Geburtshilfe e.V., DGPFG) and to collaboration partners of the CarePreg study. Because the needed sample size of *n* = 176 was not reached within the first three months after starting recruitment, we repeated the described cluster sampling method in a second round. Therefore, we additionally selected one large city, two medium-sized towns, three small towns and five rural communities from each federal state in Germany by using prompts in ChatGPT-4, resulting in *n* = 143 cities, towns and communities, this time not including the three city-states. In this second round, *n* = 831 gynecologists and gynecological practices received an e-mail followed by two reminder e-mails. Additionally, about 200 flyers with a QR code and short study description were distributed at the German Congress of Gynecology and Obstetrics (German: Kongress der Deutschen Gesellschaft für Gynäkologie und Geburtshilfe, DGGG-Kongress) in October 2024. Data collection was stopped in November 2024 when the calculated sample size of 176 participants was reached.

### Data analysis

We used IBM SPSS Statistics 29 on Windows to perform statistical analyses. Both fully completed and partially completed surveys were included in the analyses. Therefore, the sample size may vary for each construct. Participants who did not meet inclusion criteria and did not complete the demographic and professional characteristics were excluded. Based on guidelines for handling missing data [34], item-level missing values were imputed using the person mean when at least one item response was available. Construct-level analyses were conducted using all available data without further imputation, as less than 10% of values were missing. Descriptive statistics were calculated for all items and scales (see Additional file 2). Sum scores and mean values were calculated, considering the inversion of individual items.

Participants were assigned to the group ‘*providers’* if they indicated that they currently offer medical and/or surgical abortions before and/or after the 12th week of pregnancy p.c. Otherwise, they were assigned to the group of ‘*non-providers’*. TPB was tested through a multiple linear regression analysis with behavioral intention as dependent variable and attitudes, subjective norms and perceived behavioral control as independent variables [28]. We evaluated key assumptions for multiple regression, including linearity, normality of residuals, homoscedasticity, and the absence of multicollinearity among independent variables. Residuals displayed heteroscedasticity. Therefore, we applied a bootstrap procedure with 1,000 resamples to compute robust standard errors. All other assumptions were satisfied. As the TPB is a general framework and does not prescribe specific additional control variables [12], no further covariates were included in the model. T-tests and a chi-square tests were conducted to compare abortion providers and non-providers regarding their attitudes, subjective norms, perceived behavioral control, behavioral intention, fear of stigmatization, gender, religiosity, subjective knowledge on abortion and subjective knowledge on current legal regulations of abortions. For t-tests, we assessed the homogeneity of variances using Levene’s test and examined whether values were approximately normally distributed within each group. When the assumption of equal variances was violated, we report the Welch t-test, which does not assume homogeneity of variances. Slight deviations from normality were observed for some outcomes; however, given the large sample size, the central limit theorem ensures that the t-test results remain robust. Generally, p-values < 0.05 were considered as statistically significant. Given the exploratory nature of the study and the absence of predefined hypotheses regarding the joint distribution or interaction of the dependent variables, we chose a univariate analytical approach focusing on individual outcomes. A Bonferroni correction was applied to control for multiple comparisons. Therefore, for eight t-tests and one chi-square test in total, p-values of < 0.00555 were considered statistically significant [35]. We calculated effect size Cohen’s d for the t-tests and categorized them using the following thresholds: small (0.2), medium (0.5), large (0.8), and very large (1.3) [36].

## Results

### Description of the sample

In total, the data set contained *n* = 247 surveys. Of about 2.000 potential respondents, about 10% filled out the survey. *N* = 34 participants were excluded because they did not meet the inclusion criteria (*n* = 15), did not continue the survey after consenting to the data protection regulations (*n* = 10) or did not continue the survey after questions on inclusion criteria (*n* = 9). Therefore, a total of *n* = 213 surveys were included in analyses, *n* = 191 participants answered all items of the analyzed constructs. Table 2 shows demographic and professional characteristics of the sample, further participant characteristics can be found in Additional file 2 (Table A). Most of the participants (*n* = 135; 63.4%) were middle-aged (40–59 years), and *n* = 173 (81.2%) identified as female. *N* = 158 (74.2%) participants reported eleven or more years of professional experience in their specialty. All of Germany’s federal states were represented, as well as every type of city size (see Additional file 2, Table A). *N* = 212 participants (95.5%) were gynecologists and *n* = 1 (0.5%) was a general medicine physician who is providing abortions. *N* = 95 (44.6%) had no religion, followed by *n* = 75 (35.2%) Protestants, *n* = 36 (16.9%) Roman-Catholics, and *n* = 7 (3.3%) participants stated another religion.

**Table 2:** Demographic and professional characteristics (*n* = 213)

**Characteristics**	***N***	**%**
Age		
<30 years	0	0
30–39 years	17	8
40–49 years	70	32.9
50–59 years	65	30.5
≥ 60 years	61	28.6
Gender		
Male	38	17.8
Female	173	81.2
Other	1	0.5
Not reported	1	0.5
Years as specialist physician		
< 5 years	18	8.5
5–10 years	37	17.4
11–20 years	66	31.0
21–30 years	63	29.6
>31 years	29	13.6
Medical specialty		
Obstetrics and gynecology	212	95.5
General medicine^a^	1	0.5
Office-based		
Yes	208	97.7
No^a^	5	2.3
Religion		
None	95	44.6
Roman catholic	36	16.9
Protestant	75	35.2
Islam	2	0.9
Judaism	1	0.5
Other	4	1.9
Incorporating religious beliefs in life *(R1)*		
No religious identification	68	31.9
Not at all	44	20.7
A little	36	16.9
Somewhat	46	21.6
Quite a bit	15	7.0
A great deal	4	1.9
Frequency of religious attendance *(R2)*		
Never	72	33.8
Once in a year or less	50	23.5
A few times a year	82	38.5
A few times a month	9	4.2
Once a week or more	0	0

^a^This characteristic was only possible if the participant provided abortions

### Behavior

As shown in Table 3, *n* = 118 (55.4%) of the participants provided medical abortions, *n* = 54 (25.4%) provided surgical abortions before the 12th week of pregnancy p.c. and *n* = 20 (9.4%) after the 12th week of pregnancy p.c. Accordingly, the participants were divided into two groups: *n* = 120 (56.3%) provided medical and/or surgical abortion (= *providers*) and *n* = 93 (43.7%) did not provide any form of abortion (= *non-providers*). Of *n* = 120 (56.3%) abortion *providers*, *n* = 115 (54.0%) were office-based and *n* = 5 (2.3%) were not office-based (employed physicians). Regardless of providing abortions or not, *n* = 34 (16.0%) offered mandatory state-recognized pregnancy conflict counselling, which patients need for an abortion according to the counselling regulation. *N* = 146 (68.5%) stated that they offer open counselling without providing the state-recognized counselling certificate for patients with unintended pregnancies. Differences regarding offering state-recognized pregnancy conflict counselling and open counselling between *providers and non-providers* are shown in Table 3. Sixty-seven out of 93 non-providers (72.0%) stated that they refer patients to other physicians who offer abortions.

**Table 3:** Behavior regarding abortion provision and abortion care

Characteristics	Non-Providers (*n* = 93) *N* (%)	Providers (*n* = 120) *N* (%)	Total (*n* = 213) *N* (%)
Method of abortion provided^a^			
Medical abortions			
- Mifepristone and Misoprostol	-	84 (70.0)	84 (39.4)
- Home Use	-	80 (66.7)	80 (37.6)
- Telemedical offer	-	10 (8.3)	10 (4.7)
Surgical abortions			
- Vacuum aspiration < 12 weeks	-	54 (45.0)	54 (25.4)
- Curettage < 12 weeks	-	9 (7.5)	9 (4.2)
- Surgical abortion < 12 weeks	-	20 (16.7)	20 (9.4)
Offer of state-recognized pregnancy conflict counselling	10 (10.8)	24 (20.0)	34 (16.0)
Offer of open counselling for patients with unintended pregnancy	74 (20.4)	72 (60.0)	146 (68.5)
Referral to physicians who offer abortions^b^	67 (72.0)	-	67 (72.0)

^a^This item was only presented to providers: If the participant stated that they offer a medical or surgical abortion, they were asked about the methods (multiple answers possible).

^b^This item was only presented to non-providers

### Attitudes

Sum scores of the AAS for our participants ranged between 29 and 70 (on a scale from 0 to 70), accordingly no participant was classified into the categories ‘moderate contra abortion’ or ‘strong contra abortion’. Differences between *providers* and *non-providers* are shown in Table 4. With a total mean of 54.46 (SD 7.76), most participants were categorized as ‘moderate pro abortion’. In comparison, *providers* had a mean of 56.65 (SD 7.07) and were therefore ‘strong pro abortion’ on average, whereas non-providers had a mean of 51.69 (SD 7.73) and were ‘moderate pro abortion’ on average.

**Table 4:** Abortion attitude scale (AAS) (possible range 0–70)

	Sum score range	Non-providers (*n* = 91) *N* (%)	Providers (*n* = 114) *N* (%)	Total (*n* = 205) *N* (%)
Strong pro abortion	56–70	32 (34.4)	70 (58.3)	102 (47.9)
Moderate pro abortion	44–55	42 (45.2)	40 (33.3)	82 (38.5)
Unsure	43 − 27	17 (18.3)	4 (3.3)	21 (9.9)
Moderate contra abortion	16–26	0 (0)	0 (0)	0 (0)
Strong contra abortion	0–15	0 (0)	0 (0)	0 (0)

All constructs analyzed via t-tests are shown in Table 5, presenting total means, group means, t-value, p-value and Cohen’s d. The t-test revealed a statistically significant group difference regarding *attitudes* towards abortion between *providers* and *non-providers* (t(df) = -4.79 (204), p = < 0.001, d = 0.672). Therefore, abortion *providers* showed more positive *attitudes* towards abortion than *non-providers*.

**Table 5:** Means and group comparisons: variables of the TPB, religiosity, fear of stigmatization and subjective knowledge

Construct	Number of items	Possible range	Non-Providers Mean (SD) (*N*)	Providers Mean (SD) (*N*)	Total Mean (SD) (*N*)	t (df)	*p*-value	Cohen’s d
*Attitudes*	14	0–70	51.69 (7.73) (*n* = 91)	56.65 (7.07) (*n* = 115)	54.46 (7.76) (*n* = 206)	4.79 (204.0)	< 0.001	0.672
*Subjective norms*	3	1–7	2.25 (1.14) (*n* = 91)	3.40 (1.01) (*n* = 112)	2.88 (1.21) (*n* = 203)	7.60 (201.0)	< 0.001	1.072
*Perceived behavioral control*	4	1–7	4.19 (1.46) (*n* = 91)	6.07 (0.90) (*n* = 112)	5.23 (1.51) (*n* = 203)	10.70 (144.16)	< 0.001	1.581
*Behavioral Intention*	3	1–7	2.54 (1.92) (*n* = 91)	6.67 (0.86) *(n* = 112)	4.82 (2.51) (*n* = 203)	19.01 (118.81)	< 0.001	2.876
*Religiosity*	2	1–10	4.40 (2.09) (*n* = 93)	3.16 (1.94) (*n* = 120)	3.70 (2.10) (*n* = 213)	-4.47 (211)	< 0.001	0.617
*Fear of stigmatization*	10	10–50	22.15 (9.28) (*n* = 87)	17.17 (6.13) (*n* = 109)	19.38 (8.06) (*n* = 196)	-4.31 (142.44)	< 0.001	0.647
*Subjective knowledge on abortion*	2	2–12	10.21 (2.01) (*n* = 85)	11.80 (0.64) (*n* = 109)	11.10 (1.62) (*n* = 194)	7.00 (97.10)	< 0.001	1.122
*Subjective knowledge on current legal regulations of abortions in Germany*	1	1–6	5.04 (1.13) (*n* = 85)	5.61 (0.72) (*n* = 109)	5.36 (0.96) (*n* = 194)	4.06 (135.27)	< 0.001	0.619

### Subjective norms, perceived behavioral control, behavioral intention

*Subjective norms* of providing abortion were stated as rather low, with a total mean of 2.88 (SD 1.21 on a scale from 1 to 7). The *perceived behavioral control* of providing abortion was stated as rather high with a mean of 5.23 (SD 1.51) on a scale from 1 to 7. The *behavioral intention* to provide abortion had a total mean of 4.82 (SD 2.51) on a scale from 1 to 7 (see Table 5).

T-tests (see Table 5) revealed statistically significant differences between *providers* and *non-providers* regarding *subjective norms* (t(df) = 7.60 (201), p = < 0.001, d = 1.072), *perceived behavioral control* (t(df) = 10.70 (144.16), p = < 0.001, d = 1.581), and *behavioral intentions* (t(df) = 19.01 (118.81), p = < 0.001, d = 2.876). Abortion *providers* are more likely to perceive stronger *subjective norms* of providing abortion, *perceive* a higher *behavioral control* of providing abortion and have a higher *behavioral intention* to provide abortion than *non-providers*.

Multiple linear regression analysis accounted for approximately 52% of variance in behavioral intentions with R² = 0.522 and adjusted R² = 0.514. It showed that the *perceived behavioral control* of providing abortion (β = 0.479, *p* < 0.001) has the largest standardized regression weight on the behavioral intention to provide abortions, followed by *subjective norms* of providing abortion (β = 0.326, p = < 0.001) and *attitudes* towards abortion (β = 0.136, *p* = 0.010) (see Table 6).

**Table 6:** Multiple regression analysis on the theory of planned behavior (*n* = 203)

Model	*R*²	Adjusted *R*²	F	*p*(F)
Total (dependent variable: behavioral intention; independent variables: attitudes, subjective norms, perceived behavioral control)	0.522	0.514	72.345	< 0.001

**Independent variable**	**B (unstandardized regression coefficents)**	***95% bootstrap CI for B***	**ß (standardized regression coefficients)**	***p*** **-value**
Attitudes	0.044	0.15 − 0.071	0.136	0.010
Subjective norms	0.673	0.417–0.937	0.326	< 0.001
Perceived behavioral control	0.795	0.618–0.978	0.479	< 0.001

*R²* Coefficient of Determination, *F*  F-Test for overall significance, *p(F)*  *p*-value for F, *CI*  Confidence interval

### Religiosity and gender

The two items on religiosity (R1 and R2, see Table 2) were added to capture *religiosity* with a range of 1–10. The mean of religiosity was 3.70 (SD 2.10). A t-test (see Table 5) revealed a statistically significant group difference regarding *religiosity* between *providers* and *non-providers* (t(df) = -4.47 (211), p = < 0.001, d = 0.617). Therefore, abortion *providers* stated lower *religiosity* than *non-providers*. Using a chi-square test to compare *providers* and *non-providers* in terms of *gender*, no statistically significant difference was found (Pearsons chi-square = 1.964, *p* = 0.580).

### Fear of stigmatization

The Abortion Provider Stigma Scale – Subscale Disclosure Management [29] had a possible range of 10–50 points. Sum scores of the participants were between 10 and 47 with a mean of 19.38 (SD 8.06). *Non-providers* showed a higher fear of stigmatization with a mean of 22.15 (SD 9.28) compared to *providers* with a mean of 17.17 (SD 6.13), as shown in Table 6. Means of individual items are listed in Additional file 2, Table G. A t-test (see Table 5) revealed a statistically significant group difference regarding *fear of stigmatization* between *providers* and *non-providers* (t(df) = -4.31 (142.44), p = < 0.001, d = 0.647). *Non-providers* showed a higher *fear of stigmatization* than abortion *providers*.

### Subjective knowledge

Regarding the items on subjective knowledge *n* = 187 (96.3%) of all participants (99.1% of the *providers*; *92*.9% of the *non*-*providers*) stated that they had enough theoretical knowledge about abortion to be able to advise patients (agree/strongly agree to the respective item). *N* = 158 (81.5%) (98.1% of the *providers*; 60.0% of the *non-providers*) stated that they had sufficient knowledge to perform abortions. *N* = 165 (85.1%) (89.9% of the *providers*; 78.8% of the *non-providers*) stated that they know the World Health Organization’s recommendations [5] for performing abortions, and *n* = 163 (84.0%) (94.5% of the *providers*; 70.6% of the *non-providers*) the current German S2k guidelines [6] on performing abortions. Regarding the legal regulations and laws on abortion in Germany, *n* = 168 (86.6%) (95.4% of the *providers*; 75.3% of the *non-providers*) stated that they considered themselves to be well informed. All responses for subjective knowledge in total and group comparison are found in Additional file 2, Table H. Two items about knowledge on abortion (“I have sufficient theoretical knowledge of abortion to be able to advise patients on this topic/to perform an abortion in practice”) were added up to assess general *subjective knowledge on abortion*. On a scale from 2 to 12, there was a total mean of 11.10 (SD 1.62), which indicates a high perceived knowledge. The *subjective knowledge on current legal regulation of abortions* had a mean of 5.36 (SD 0.96) on a scale from 1 to 6. T-tests (see Table 5) revealed statistically significant group differences between *providers* and *non-providers* regarding *subjective knowledge on abortion* (t(df) = 7.00 (97.10), p = < 0.001, d = 1.122) and *subjective knowledge on current legal regulations of abortion* (t(df) = 4.06 (135.27), p = < 0.001, d = 0.619). Abortion *providers* rated their *knowledge on abortion* as well as *on current legal regulations of abortions* in Germany better than *non-providers*.

Further results on attitudes, reasons for non-provision, questions about the abolition of § 219a, fear of criminalization, questions on availability of abortion care, questions about education as well as objective knowledge on abortion can be found in Additional file 2.

## Discussion

This study applied the TPB to examine the factors associated with physicians’ intentions to provide abortion care in Germany. Specifically, we analyzed the influence of *attitudes* toward abortion, *subjective norms* regarding abortion provision, and *perceived behavioral control* on this intention. Additionally, we examined potential influencing factors identified in previous research, including *gender*,* religiosity*,* fear of stigmatization*, and *subjective knowledge*, through a group comparison between abortion providers and non-providers. Combining a theory-based framework with additional contextual factors, thereby, provides new insights into possible determinants of intention to provide abortions in Germany.

Group comparisons revealed that abortion providers have more positive *attitudes* towards abortions, stronger *subjective norms*, higher *perceived behavioral control*, higher *behavioral intention* to provide abortions, lower *fear of stigmatization*, lower *religiosity* and higher *subjective knowledge on abortions and abortion laws*. No significant difference regarding gender was found. The strongest effect sizes were found in *subjective norms*,* perceived behavioral control*,* behavioral intention* and *subjective knowledge* on abortions. According to the multiple regression analysis of the TPB, the strongest association with *behavioral intention* could be found for *perceived behavioral control*, followed by *subjective norms* and *attitudes.*

Previous international studies on compliance to guidelines on abortion care [37], intention to seek abortion training and provide abortion care in the future [38], as well as studies applying the TPB in other contexts (e.g., referral of cancer patients to psychosocial support [14], providing cervical cancer screening [13]), showed *attitudes* and *subjective norms* as the most influential factors on the *behavioral intention*. Myran et al. (2015) found that medicals students’ *attitudes towards abortion training and provision* is the strongest predictor of their intention to seek abortion training and to provide abortion services in the future. Kim and Steinberg (2023) have shown via an online-survey of 1,106 women, that interpersonal contact helps to improve abortion *attitudes* [39]. Especially knowing someone who had an abortion or having an abortion oneself was associated with more positive *attitudes* and accurate knowledge. Turner et al. (2018) tested a “Value clarification and attitude transformation (VCAT) workshop” for *abortion providers*, trainers and policymakers in 12 countries [40]. After the workshop, participants showed increased *knowledge*, *attitudes* and *behavioral intentions* related to abortion care.

However, in our study p*erceived behavioral control* was surprisingly shown to be the strongest influencing factor on the *intention* to provide abortion care. Given that both abortion providers and non-providers had positive overall attitudes toward abortion, attitudes may only have a limited impact within our data. A lower *perceived behavioral control* of non-providers indicates that they might not feel able to provide abortions or might perceive themselves as lacking control over the decision to provide abortions. Ajzen (1991) describes that perceived behavioral control is dependent on control beliefs, which are formed by resources and opportunities available to a person, the confidence in their ability to perform it and the actual feeling of control [12]. As Maeffert et al. (2023) [10] describe, high bureaucratic barriers exist for offering medical abortions because of the distribution law for drugs used for abortion (§47a “Arzneimittelgesetz”) and a complex billing process for abortion services (§24b SGB V). However, from our data, we cannot draw conclusions on the influence of abortion laws, directives from employers, bureaucratic barriers or lack of confidence and resources on the influence of *perceived behavioral control* on intention to provide abortions in Germany.

In line with previous studies, our study showed that abortion *providers* showed lower *religiosity* than *non-providers* [41–43]. Religious personal environment and fear of stigmatization by the personal environment were already described as a barrier to (intention of) abortion provision [15]. Schmuhl et al. (2021) showed in a survey of 1,357 physicians that low religiosity is associated with a higher willingness to consult in abortion-related cases [18]. Farmer et al. (2022) showed in their survey of 162 medical students that among the participants who were unlikely to provide medical or surgical abortions around 42–55% stated religious objections as one of the reasons that prevents them from including abortion in their future practice [19].

In accordance with previous studies, *non-providers* showed a higher *fear of stigmatization* than *providers* [16, 17, 23]. Martin et al. (2014) described that their “Providers Share Workshops”, a safe space for abortion providers to discuss their work experiences, could decrease stigma of participants [44]. They also described that higher stigma predicts lower compassion satisfaction, higher burnout and higher compassion fatigue [44].

Regarding *subjective knowledge on abortion* and *knowledge on current legal regulations of abortions*, a U.S. survey of 2,149 obstetrician-gynecologists by Steinauer et al. (2008) showed that the availability of abortion training during residency was positively correlated with providing abortions in later practice, regardless of intention to provide abortion before residency [45]. Pace et al. (2008) have shown that the ‘Medical Students for Choice’s Reproductive Health Externship program’ can positively impact medical students’ knowledge, attitudes and intentions to provide abortions [46]. Hence, the lack of knowledge on abortion and legal regulations in our sample might be due to the fact that abortion was not a mandatory part of residency training until 2025 in Germany and is still not implemented in all medical school curricula [47]. Thus, many practicing physicians did not receive theoretical or practical training during their education. Although education courses are available outside from medical schools, participation is voluntary and must be organized independently.

### Strengths and limitations

This study has examined a variety of possible influencing factors on the intention to provide abortions as a physician in Germany in an exploratory approach and has applied the TPB on abortion provision for the first time. During the recruitment process we were able to reach all federal states and city sizes to achieve a heterogeneous study population. With a percentage of 81.2% female physicians, we had a good representation of gender compared to the actual percentage of female office-based gynecologists in Germany (71.9% in 2022 [48]). However, our sample might be characterized by a selection bias.

City selection was performed using ChatGPT-4, which is not a randomization tool. As a large language model, it generates outputs based on probabilistic patterns rather than genuine randomness. Therefore, the selected cities cannot be considered a random sample. This may introduce selection bias and limits the generalizability of the findings, as the sample may not accurately represent the broader population of locations.

Furthermore, due to the additional recruitment via the “Doctors for Choice Germany” network later in the course of the study, some participants may took part in the study because they had a particular interest in the topic. This assumption of a selective sample is underlined by the overall very positive attitudes of the included sample. Furthermore, a potential influence of social desirable answers should be taken into account even though the survey was designed to ensure anonymity [49]. In our analyses we applied validated scales (AAS for assessing *attitudes*, APSS for assessing *fear of stigmatization*), adapted items (assessing *subjective norms*,* perceived behavioral control*,* behavioral intention*,* religiosity*) and self-developed items (assessing *subjective knowledge*). Adapted and self-developed items had not undergone formal validation which may reduce internal validity of the study, as measurement imprecision could have influenced the magnitude of the observed associations. Accordingly, results based on sum scores and group differences derived from self-developed and non-validated items should be interpreted with particular caution. Furthermore, with the AAS we assessed *attitudes towards abortion* whereas influences of *subjective norms*,* perceived behavioral control* on *intention to participate in abortion care* was assessed. Future research should develop well-validated instruments for constructs like *subjective knowledge* about abortion and *attitudes* towards abortion provision and investigate whether the results of our exploratory analyses can be confirmed with those instruments.

Another limitation of this study is the use of multiple univariate analyses rather than a multivariate approach. While this decision was appropriate given the exploratory nature of the study, no a priori hypotheses regarding the interrelationships among the dependent variables were formulated. Future research guided by theory-driven hypotheses could employ multivariate approaches to further investigate interrelationships and joint effects among factors influencing the intentions to participate in abortion service provision.

## Conclusions

The results of this study show that there are relevant influencing factors on the intention to provide abortions, such as *attitudes*, *subjective norms* and *perceived behavioral control*, as well as differences between *providers* and *non-providers* on the *variables of the TPB*, *religiosity*, *fear of stigmatization* and *knowledge* about abortion. Some of the correlations observed could already be surmised on the basis of international literature on abortion care. Given the significant group differences in *providers* and *non-providers* and the substantial influence of *perceived behavioral control* on *behavioral intention* found in our data, further research on perceived behavioral control in the context of abortion provision in Germany and multivariate approaches to identify the influences of for example *stigma* and *lack of knowledge* are warranted. In future research, interventions should be developed which help physicians to feel safe, able and accepted to offer abortions. Those should focus particularly on modifiable factors that may act as barriers to abortion provision as identified in this study.

## Supplementary Information

Supplementary Material 1. Additional File 1: CHERRIES-Checklist.

Supplementary Material 2. Additional File 2: (Additional) Descriptive results of all constructs of the survey.

Supplementary Material 3. Additional File 3: Details on analyzed constructs.

Supplementary Material 4. Additional File 4: Survey in its original language.

## Data Availability

The datasets used and/or analyzed during the current study are available from the corresponding author on reasonable request.

## Abbreviations

AAS: Abortion Attitude Scale
AKF: Arbeitskreis Frauengesundheit in Medizin, Psychotherapie und Gesellschaft
APSS: Abortion Provider Stigma Scale
BMG: Bundesministerium für Gesundheit
CHERRIES: Checklist for Reporting Results of Internet E-Surveys
DGGG: Deutsche Gesellschaft für Gynäkologie und Geburtshilfe
DGPFG: Deutsche Gesellschaft für Psychosomatische Frauenheilkunde und Geburtshilfe
p.c.: Post conceptionem
p.m.: Post menstruationem
SchKG: Schwangerschaftskonfliktgesetz
StGB: Strafgesetzbuch
TPB: Theory of Planned Behavior
TRAPD: Translation, Review, Adjudication, Pretest and Documentation
WHO: World Health Organization
