# Supplementary material for: Barriers and motivations: analyzing the factors influencing abortion provision by gynecologists in Germany – a cross-sectional study
Source: Reprod Health. 2026 Jun 18;23:125. doi: 10.1186/s12978-026-02389-8 (PMC13288800; doi:10.1186/s12978-026-02389-8)
Supplement: Supplementary file 2 — Supplementary Material 2. Additional File 2: (Additional) Descriptive results of all constructs of the survey. [file 12978_2026_2389_MOESM2_ESM.docx]

## Additional file 2: (Additional) Descriptive results of all constructs of the survey

| **Table A: Demographic characteristics (*n*=213)** | |  |
| --- | --- | --- |
| **Characteristics** | N | % |
| Federal state of **working place** |  |  |
| Baden-Württemberg | 17 | 8.0 |
| Bavaria | 8 | 3.8 |
| Berlin | 30 | 14.1 |
| Brandenburg | 9 | 4.2 |
| Bremen | 9 | 4.2 |
| Hamburg | 22 | 10.3 |
| Hesse | 13 | 6.1 |
| Mecklenburg-Vorpommern | 12 | 5.6 |
| Niedersachsen | 12 | 5.6 |
| North Rhine-Westphalia | 24 | 11.3 |
| Rhinland-Pfalz | 5 | 2.3 |
| Saarland | 4 | 1.9 |
| Sachsen | 19 | 8.9 |
| Sachsen-Anhalt | 7 | 3.3 |
| Schleswig-Holstein | 16 | 7.5 |
| Thuringia | 6 | 2.8 |
| Town size of **working place** |  |  |
| Large city (more than 100.000 inhabitants) | 138 | 64.8 |
| Medium-sized town (20.000-100.000 inhabitants) | 52 | 24.4 |
| Small town (5.000-20.000 inhabitants) | 22 | 10.3 |
| Rural community (less than 5.000 inhabitants) | 1 | 0.5 |
| Federal state of **childhood and youth** |  |  |
| Baden-Württemberg | 24 | 11.3 |
| Bavaria | 10 | 4.7 |
| Berlin | 13 | 6.1 |
| Brandenburg | 11 | 5.2 |
| Bremen | 3 | 1.4 |
| Hamburg | 11 | 5.2 |
| Hesse | 10 | 4.7 |
| Mecklenburg-Vorpommern | 14 | 6.6 |
| Niedersachsen | 24 | 11.3 |
| North Rhine-Westphalia | 26 | 12.2 |
| Rhinland-Pfalz | 7 | 3.3 |
| Saarland | 2 | 0.9 |
| Sachsen | 20 | 9.4 |
| Sachsen-Anhalt | 7 | 3.3 |
| Schleswig-Holstein | 18 | 8.5 |
| Thuringia | 4 | 1.9 |
| Did not grow up in Germany | 9 | 4.2 |
| Town size of the **place of childhood and youth** |  |  |
| Large city (more than 100.000 inhabitants) | 90 | 42.3 |
| Medium-sized town (20.000-100.000 inhabitants) | 54 | 25.4 |
| Small town (5.000-20.000 inhabitants) | 38 | 17.8 |
| Rural community (less than 5.000 inhabitants) | 31 | 14.6 |

| **Table B: Reasons for non-provision of abortionª (*n*=211)** (multiple answers possible) | | |
| --- | --- | --- |
| **Reason** | **Non-Providers (*n*=91) *N* (%)** | **Providers (*n*=120) *N* (%)** |
| Remuneration too low | 8 (8.8) | 59 (50.8) |
| Ethical/moral reasons | 22 (24.2) | 71 (59.2) |
| Religious reasons | 6 (6.6) | 38 (31.7) |
| Fear of stigmatization | 4 (4.4) | 79 (65.8) |
| Fear of harassment/threat | 5 (5.5) | 61 (50.8) |
| The service is too complex and costly | 29 (31.9) | 48 (40) |
| Lack of practical experience | 23 (25.3) | 75 (62.5) |
| Uncertainty about the procedure | 9 (9.9) | 68 (56.7) |
| Staff do not want this | 5 (5.5) | 13 (10.8) |
| *ªProviders: Why do you think many gynecologists do not offer abortions? (multiple answers possible)*  *Non-Providers: Why do you not offer abortions? (multiple answers possible)* | | |

| **Table C: Questions about §219a (*n*=211)** | | | | | | |
| --- | --- | --- | --- | --- | --- | --- |
| **Questions about information provision on the own practice website depending on the abolition of §219a** | | | | | | |
|  | **Non-Providers (*n*=91)  *N* (%)** | **Providers (*n*=120) *N* (%)** | | | **Total (*n*=211) *N* (%)** | |
| Information on abortion on the website **before** the abolition of §219a |  |  | | |  | |
| Yes | 3 (3.3) | 17 (14.2) | | | 20 (9.5) | |
| No | 74 (81.3) | 88 (73.3) | | | 162 (76.8) | |
| I do not have a website | 14 (15.4) | 15 (12.5) | | | 29 (13.7) | |
| Information on abortion on the website **after** the abolition of §219a |  |  | | |  | |
| Yes | 6 (6.6) | 38 (31.7) | | | 44 (20.9) | |
| No | 71 (78.0) | 67 (55.8) | | | 138 (65.4) | |
| I do not have a website | 14 (15.4) | 15 (12.5) | | | 29 (13.7) | |
| **Questions for those participants, who provided information on their website (providers & non-providers)** (multiple answers possible) | | | | | | |
|  | **Before the abolition of §219a (*n*=20)  *N* (%)** | | **After the abolition of §219a (*n*=44) *N* (%)** | | | |
| Information are provided about… |  | |  | | | |
| Methods of abortion | 14 (70) | | 31 (70-5) | | | |
| Offer of abortion | 11 (55) | | 32 (72.7) | | | |
| Consulting process | 5 (25) | | 16 (36.4) | | | |
| Addresses of counseling centers | 6 (30) | | 18 (40.9) | | | |
| Links to information about abortion | 5 (25) | | 15 (34.1) | | | |
| **Table D: Abortion Attitude Scale (*n*=206)** (0 = ‘strongly disagree’ to 5 = ‘strongly agree’) | | | | | |  |
| Items | Non-providers (n=91) N (%) | Providers (n=114-115) N (%) | | Total (n=206) Mean (SD) | |  |
| The supreme court should strike down legal abortions in Germany.ª | 0.25 (0.94) | 0.12 (0.58) | | 0.18 (0.76) | |  |
| Abortion is a good way of solving an unwanted pregnancy. | 3.90 (1.32) | 4.48 (0.89) | | 4.22 (1.13) | |  |
| A mother should feel obligated to bear a child she has conceived.ª | 0.63 (1.03) | 0.33 (0.69) | | 0.46 (0.86) | |  |
| Abortion is wrong no matter what the circumstances are. ª | 0.26 (0.59) | 0.10 (0.35) | | 0.17 (0.48) | |  |
| A fetus is not a person until it can live outside its mother’s body. | 1.70 (1.52) | 2.60 (1.58) | | 2.20 (1.61) | |  |
| The decision to have an abortion should be the pregnant mother’s. | 4.56 (0.67) | 4.68 (0.73) | | 4.63 (0.71) | |  |
| Every conceived child has the right to be born. ª | 1.57 (1.38) | 0.99 (1.20) | | 1.25 (1.31) | |  |
| A pregnant female not wanting to have a child should be encouraged to have an abortion. | 1.96 (1.24) | 1.99 (1.27) | | 1.98 (1.26) | |  |
| Abortion should be considered killing a person. ª | 0.66 (0.97) | 0.19 (0.66) | | 0.40 (0.84) | |  |
| People should not look down on those who choose to have abortions. | 4.64 (0.89) | 4.68 (0.98) | | 4.66 (0.94) | |  |
| Abortion should be an available alternative for unmarried, pregnant teenagers. | 3.45 (1.46) | 4.10 (1.26) | | 3.81 (1.29) | |  |
| Persons should not have the power over the life or death of a fetus. ª | 1.08 (1.21) | 0.93 (1.23) | | 1.00 (1.22) | |  |
| Unwanted children should not be brought into the world. | 2.15 (1.30) | 2.52 (1.36) | | 2.36 (1.34) | |  |
| A fetus should be considered a person at the moment of conception. ª | 1.22 (1.91) | 0.78 (1.14) | | 0.98 (1.18) | |  |
| *ªThis item was inverted for sum scores* |  |  | |  | |  |

| **Table E: Attitudes: Approval or rejection of […] (*n*=206)** | | | |
| --- | --- | --- | --- |
|  | **Non-providers (*n*=91) *N* (%)** | **Providers (*n*=115) *N* (%)** | **Total (*n*=206) *N* (%)** |
| Medication abortion |  |  |  |
| Strongly oppose | 0 (0) | 0 (0) | 0 (0) |
| Somewhat oppose | 2 (2.2) | 0 (0) | 2 (1) |
| Neither oppose nor support | 10 (11) | 2 (1.7) | 12 (5.8) |
| Somewhat support | 24 (26) | 8 (7) | 32 (15.5) |
| Strongly support | 55 (60.4) | 105 (91.3) | 160 (77.7) |
| Surgical abortion |  |  |  |
| Strongly oppose | 0 (0) | 0 (0) | 0 (0) |
| Somewhat oppose | 1 (1.1) | 2 (1.7) | 3 (1.5) |
| Neither oppose nor support | 10 (11) | 4 (3.5) | 14 (6.8) |
| Somewhat support | 30 (33) | 35 (30.4) | 65 (31.6) |
| Strongly support | 50 (54.9) | 74 (64.3) | 124 (60.2) |
| Mandatory counseling before abortion |  |  |  |
| Strongly oppose | 2 (2.2) | 12 (10.4) | 14 (6.8) |
| Somewhat oppose | 3 (3.3) | 11 (9.6) | 14 (6.8) |
| Neither oppose nor support | 3 (3.3) | 12 (10.4) | 15 (7.3) |
| Somewhat support | 11 (12.1) | 25 (21.7) | 36 (17.5) |
| Strongly support | 72 (79.1) | 55 (47.8) | 127 (61.7) |
| Mandatory waiting period between counseling and abortion |  |  |  |
| Strongly oppose | 2 (2.2) | 21 (18.3) | 23 (11.2) |
| Somewhat oppose | 8 (8.8) | 12 (10.4) | 20 (9.7) |
| Neither oppose nor support | 1 (1.1) | 9 (7.8) | 10 (4.9) |
| Somewhat support | 15 (16.5) | 20 (17.4) | 35 (17) |
| Strongly support | 65 (71.4) | 46.1) | 118 (57.3) |
| Influence of physcian’s personal values on counseling and treatment |  |  |  |
| Strongly oppose | 60 (65.9) | 80 (69.6) | 140 (68) |
| Somewhat oppose | 13 (14.3) | 17 (14.8) | 30 (14.6) |
| Neither oppose nor support | 12 (13.2) | 12 (10.4) | 24 (11.7) |
| Somewhat support | 4 (4.4) | 4 (3.5) | 8 (3.9) |
| Strongly support | 2 (2.2) | 2 (1.7) | 4 (1.9) |
| Right to refuse to perform abortions as a physician |  |  |  |
| Strongly oppose | 2 (2.2) | 6 (5.2) | 8 (3.9) |
| Somewhat oppose | 1 (1.1) | 9 (7.8) | 10 (4.9) |
| Neither oppose nor support | 7 (7.7.) | 13 (11.3) | 20 (9.7) |
| Somewhat support | 12 (13.2) | 29 (25.2) | 41 (19.9) |
| Strongly support | 69 (75.8) | 58 (50.4) | 127 (61.7) |

| **Table F: Subjective norms, perceived behavioral control and behavioral intentions (*n*=203)** | | | | | |
| --- | --- | --- | --- | --- | --- |
| Items | Non-Providers (n=91)  Mean (SD) | | Providers (n =112) Mean (SD) | Total (n =203) Mean (SD) | |
| **Subjective norms** |  |  | | |  |
| Most people who are important to me think that I […] offer abortions. ª | 2.97 (1.703) | | 5.460 (1.442) | 4.42 (2.039) | |
| I am expected to offer abortions. ᵇ | 1.95 (1.573) | | 2.98 (2.214) | 2.52 (2.016) | |
| I feel social pressure to offer abortions. ᵇ | 1.82 (1.532) | | 1.61 (1.226) | 1.70 (1.372) | |
| **Perceived behavioral control** |  | |  |  | |
| I am confident that I could offer abortions. ᵇ | 3.69 (2.259) | | 6.25 (1.614) | 5.10 (2.309) | |
| For me it is [...] to offer abortions. ᶜ,ᵈ | 4.95 (1.911) | | 2.04 (1.328) | 3.34 (2.168) | |
| The decision to provide abortions is in my control. ᵇ | 5.66 (2.104) | | 6.74 (0.744) | 6.26 (1.602) | |
| Whether I provide abortions is not entirely up to me. ᵇ,ᵈ | 3.65 (2.579) | | 2.69 (2.360) | 3.12 (2.501) | |
| **Behavioral intention** |  | |  |  | |
| I expect to (also) provide abortions in the future. ᵇ | 2.54 (1.945) | | 6.72 (0.762) | 5.85 (2.521) | |
| I want to provide abortions in the future. ᵇ | 2.68 (2.070) | | 6.62 (1.032) | 4.56 (2.522) | |
| I intend to provide abortions in the future. ᵇ | 2.40 (1.908) | | 6.65 (1.029) | 4.74 (2.590) | |
| ª 1 ‘definitely should not’ – 7 ‘definitely should’ ᵇ 1 ‘strongly disagree’ – 7 ‘strongly agree’  ᶜ 1 ‘easy’ – 7 ‘difficult’ | | | | | |

| **Table G: Fear of stigmatization: Abortion Provider Stigma Scale – Subscale Disclosure Management** **ª (*n*=196)** (Each item: 1 = ‘never’, 2 = ‘rarely’, 3 = ‘sometimes’, 4 = ‘often’, 5 = ‘always’) | | | |
| --- | --- | --- | --- |
| Items | Non-Providers (n=87)  Mean (SD) | Providers (n =109) Mean (SD) | Total (n =196) Mean (SD) |
| People’s reactions to my being an abortion worker (would) make me keep to myself. | 2.69 (1.184) | 2.29 (0.946) | 2.47 (1.074) |
| I feel like if I (would) tell people about my work they will only see me as an abortion worker. | 2.14 (0.942) | 1.61 (0.827) | 1.85 (0.915) |
| I (would) worry about telling people I work in abortion care. | 2.41 (1.126) | 1.80 (0.911) | 2.07 (1.055) |
| It (would) bother(s) me if people in my neighborhood know that I work in abortion care. | 2.00 (1.151) | 1.61 (0.891) | 1.79 (1.030) |
| I (would) avoid telling people what I do for a living. | 2.54 (1.328) | 1.65 (0.809) | 2.05 (1.156) |
| I (would be) am afraid that if I tell people I work in abortion care I could put myself, or my loved ones, at risk for violence. | 2.30 (1.231) | 1.69 (0.778) | 1.96 (1.047) |
| I (would) feel that disclosing my abortion work is not worth the potential hassle that could result. | 2.38 (1.269) | 1.94 (1.035) | 2.14 (1.162) |
| I (would be) am afraid of how people will react if they find out about my work in abortion care. | 2.09 (1.052) | 1.72 (0.914) | 1.88 (0.993) |
| I (would) feel the need to hide my work in abortion care from my friends. | 1.43 (0.757) | 1.25 (0.596) | 1.33 (0.676) |
| I (would) find it hard to tell people I work in abortion care. | 2.17 (1.048) | 1.61 (0.817) | 1.89 (0.966) |
|  |  |  |  |
| **Fear of criminalization (**n**=196)** |  |  |  |
| I (would be) am afraid of criminalization through the current legal regulation of abortion. | 2.01 (1.185) | 1.97 (1.176) | 2.05 (1.197) |
| *ªThe providers were asked about their actual fear, while the non-providers were asked about their anticipated fear* | | | |

| **Table H: Subjective knowledge (*n*=194)** | | | |
| --- | --- | --- | --- |
|  | **Non-Providers (*n*=85) N (%)** | **Providers (*n*=109) N (%)** | **Total (*n*=194) N (%)** |
| I have sufficient theoretical knowledge of abortion to be able to advise patients on this topic. |  |  |  |
| Strongly agree | 59 (69.4) | 99 (90.8) | 158 (81.4) |
| Agree | 20 (23.5) | 9 (8.3) | 29 (14.9) |
| Somewhat agree | 3 (3.5) | 1 (0.9) | 4 (2.1) |
| Somewhat disagree | 1 (1.2) | 0 (0) | 1 (0.5) |
| Disagree | 1 (1.2) | 0 (0) | 1 (0.5) |
| Strongly disagree | 1 (1.2) | 0 (0) | 1 (0.5) |
| I have sufficient theoretical and practical knowledge of abortion to carry it out in practice. |  |  |  |
| Strongly agree | 33 (38.8) | 100 (91.7) | 133 (68.6) |
| Agree | 18 (21.2) | 7 (6.4) | 25 (12.9) |
| Somewhat agree | 20 (23.5) | 2 (1.8) | 22 (11.3) |
| Somewhat disagree | 4 (4.7) | 0 (0) | 4 (2.1) |
| Disagree | 6 (7.1) | 0 (0) | 6 (3.1) |
| Strongly disagree | 4 (4.7) | 0 (0) | 4 (2.1) |
| I know the contents of the S2k guideline (2023) on abortion in the first trimester. |  |  |  |
| Strongly agree | 34 (40.0) | 81 (74.3) | 115 (59.3) |
| Agree | 26 (30.6) | 22 (20.2) | 48 (24.7) |
| Somewhat agree | 12 (14.1) | 3 (2.5) | 15 (7.7) |
| Somewhat disagree | 5 (5.9) | 1 (0.9) | 6 (3.1) |
| Disagree | 7 (8.2) | 2 (1.7) | 9 (4.6) |
| Strongly disagree | 1 (1.2) | 0 (0) | 1 (0.5) |
| I know which methods of abortion are recommended by the WHO. |  |  |  |
| Strongly agree | 34 (40.0) | 80 (73.4) | 114 (61.3) |
| Agree | 33 (38.8) | 18 (16.5) | 51 (26.3) |
| Somewhat agree | 10 (11.8) | 7 (6.4) | 17 (8.8) |
| Somewhat disagree | 2 (2.4) | 2 (1.8) | 4 (2.1) |
| Disagree | 5 (5.9) | 2 (1.8) | 7 (3.6) |
| Strongly disagree | 1 (1.2) | 0 (0) | 1 (0.5) |
| I know where I can learn how to perform/offer abortions. |  |  |  |
| Strongly agree | 44 (51.8) | 75 (68.8) | 119 (61.3) |
| Agree | 23 (27.1) | 17 (15.6) | 40 (20.6) |
| Somewhat agree | 5 (5.9) | 4 (3.7) | 9 (4.6) |
| Somewhat disagree | 2 (2.4) | 7 (5.8) | 9 (4.6) |
| Disagree | 5 (5.9) | 6 (5.5) | 11 (5.7) |
| Strongly disagree | 6 (7.1) | 0 (0) | 6 (3.1) |
| I consider myself well informed about the state laws and regulations on abortion. |  |  |  |
| Strongly agree | 37 (43.5) | 75 (68.8) | 112 (57.7) |
| Agree | 27 (31.8) | 29 (26.6) | 56 (28.9) |
| Somewhat agree | 12 (14.1) | 3 (2.5) | 15 (7.7) |
| Somewhat disagree | 6 (7.1) | 0 (0) | 6 (3.1) |
| Disagree | 2 (2.4) | 2 (1.8) | 4 (2.1) |
| Strongly disagree | 1 (1.2) | 0 (0) | 1 (0.5) |

| **Table I: Contact during training** | | | |
| --- | --- | --- | --- |
| **Desire to learn more about […] (*n*=193)** | | | |
|  | **Non-providers (*n*=84) *N* (%)** | **Providers (*n*=109) *N* (%)** | **Total (*n*=193) *N* (%)** |
| Desire to learn more about abortion during studies |  |  |  |
| Yes | 34 (40.5) | 69 (63.3) | 103 (53.4) |
| Desire to learn more about abortion during residency |  |  |  |
| Yes | 42 (50) | 62 (56.9) | 104 (53.9) |
| **Methods that were teached during residency (*n*=194)** | | | |
|  | **Non-providers (n=85) *N* (%)** | **Providers (*n*=109) *N* (%)** | **Total (*n*=194) *N* (%)** |
| Learned the performance of induced medication abortion during residency |  |  |  |
| Yes | 30 (35.3) | 27 (24.8) | 57 (29.4) |
| Learned the performance of vacuum aspiration for induced abortion during residency |  |  |  |
| Yes | 57 (67.1) | 71 (65.1) | 128 (66.0) |
| Learned the performance of curettage for induced abortion during residency |  |  |  |
| Yes | 69 (81.2) | 73 (67.0) | 142 (73.2) |
| Learned no method of induced abortion during residency |  |  |  |
| Yes | 13 (15.3) | 21 (19.3) | 34 (17.5) |

| **Table J: Perception on the current care situation (*n*=194)** | | | |
| --- | --- | --- | --- |
|  | **Non-providers (*n*=85)  *N* (%)** | **Providers (*n*=109)  *N* (%)** | **Total (*n*=194) *N* (%)** |
| There are sufficient facilities in Germany that perform abortions. |  |  |  |
| Yes | 11 (12.9) | 14 (12.8) | 25 (12.9) |
| Rather yes | 21 (28.2) | 11 (10.1) | 35 (18.0) |
| Unsure | 12 (14.1) | 18 (16.5) | 30 (15.5) |
| Rather no | 25 (29.4) | 37 (33.9) | 62 (32.0) |
| No | 13 (15.3) | 29 (26.6) | 42 (21.6) |
| Unintended pregnant women have easy access to abortions in Germany. |  |  |  |
| Yes | 21 (24.7) | 13 (11.9) | 34 (17.5) |
| Rather yes | 28 (30.1) | 26 (23.9) | 54 (27.8) |
| Unsure | 11 (12.9) | 23 (21.1) | 34 (17.5) |
| Rather no | 16 (18.8) | 35 (32.1) | 51 (26.3) |
| No | 9 (10.6) | 12 (11.0) | 21 (10.8) |
| Unintended pregnant women in Germany have easy access to information on abortions |  |  |  |
| Yes | 31 (36.5) | 32 (29.4) | 63 (32.5) |
| Rather yes | 33 (38.8) | 45 (41.3) | 78 (40.2) |
| Unsure | 14 (16.5) | 13 (11.9) | 27 (13.9) |
| Rather no | 5 (5.9) | 14 (12.8) | 19 (9.8) |
| No | 2 (2.4) | 5 (4.6) | 7 (3.6) |
| Access to contraceptives in Germany is easy |  |  |  |
| Yes | 61 (71.8) | 60 (55.0) | 121 (62.4) |
| Rather yes | 18 (21.2) | 34 (31.2) | 52 (26.8) |
| Unsure | 1 (1.2) | 5 (4.6) | 6 (3.1) |
| Rather no | 4 (4.7) | 7 (6.4) | 11 (5.7) |
| No | 1 (1.2) | 3 (2.8) | 4 (2.1) |

| **Table K: Objective knowledge (*n*=191)**  Answer options: ‘Yes’, ‘Rather yes’, ‘Unsure’, ‘Rather no’ and ‘No’. | | | | | | |
| --- | --- | --- | --- | --- | --- | --- |
|  | **Non-providers (*n*=83)** | | **Providers (*n*=108)** | | **Total (*n*=191)** | |
|  | **Correct  *N* (%)** | **Incorrect *N* (%)** | **Correct  *N* (%)** | **Incorrect *N* (%)** | **Correct  *N* (%)** | **Incorrect *N* (%)** |
| Institutions may refuse to perform abortions. *(correct)* | 79 (95.2) | 4 (4.8) | 99 (91.7) | 9 (8.3) | 178 (93.2) | 12 (6.8) |
| Any physician may refuse to perform abortions. *(correct)* | 82 (98.8) | 1 (1.2) | 106 (98.1) | 2 (1.9) | 188 (98.4) | 3 (1.6) |
| There is an obligation for pregnant women who wish to have an abortion to undergo counseling. *(correct)* | 81 (100) | 0 (0) | 106 (98.1) | 2 (1.7) | 189 (99.0) | 2 (1.0) |
| After the consultation, the unwanted pregnant woman can have the abortion performed the very next day. *(incorrect*) | 81 (97.6) | 2 (2.4) | 103 (95.4) | 5 (4.6) | 184 (96.3) | 7 (3.7) |
| The costs for the abortion (according to counseling regulations) are generally covered by health insurance*. (incorrect)* | 80 (96.4) | 3 (3.6) | 100 (92.6) | 8 (7.4) | 180 (94.2) | 11 (5.8) |
| Abortions are associated with an increased risk of breast cancer*. (incorrect)* | 82 (98.1) | 1 (1.2) | 107 (99.1) | 1 (0.9) | 189 (99.0) | 2 (1.0) |
| Abortions almost always have negative psychological effects. *(incorrect)* | 54 (65.1) | 29 (34.9) | 88 (81.5) | 20 (18.5) | 142 (74.3) | 49 (25.7) |
| Abortions make it more difficult to become pregnant in the future. *(incorrect)* | 75 (90.4) | 8 (9.6) | 106 (98.1) | 3 (1.9) | 181 (94.8) | 10 (5.2) |
| Abortions increase the risk of future miscarriages. *(incorrect)* | 74 (10.8) | 9 (10.8) | 102 (94.4) | 6 (5.6) | 176 (92.1) | 15 (7.9) |
| *Correct statements were rated as correct by the answers ‘yes’ and ‘rather yes’, incorrect statements were rated as correct knowledge by the answer ‘no’, ‘rather no’ and ‘unsure’* | | | | | | |
