# Supplementary material for: Barriers and motivations: analyzing the factors influencing abortion provision by gynecologists in Germany – a cross-sectional study
Source: Reprod Health. 2026 Jun 18;23:125. doi: 10.1186/s12978-026-02389-8 (PMC13288800; doi:10.1186/s12978-026-02389-8)
Supplement: Supplementary file 3 — Supplementary Material 3. Additional File 3: Details on analyzed constructs. [file 12978_2026_2389_MOESM3_ESM.docx]

## Additional File 3: Details on analyzed constructs

The survey contained items regarding behavior (abortion provision) such as ‘I currently offer medical abortions/surgical abortions <12 weeks of pregnancy’ (yes/no). When participants answered ‘yes’ they were also asked which method they performed, such as for medical abortion ‘Mifepristone and Misoprostol’, ‘Home use’ which means patients can take the medical at home and ‘telemedical offer’ which means the physicians also offer calls to accompany their patients during the procedure. For the following survey items, the survey was partially divided into two different versions based on behavior. Accordingly, the Abortion Provider Stigma Scale and a question on behavioral intention, were slightly adapted to the behavior of providing or not providing abortions. Some questions, such as: ‘How long have you been providing abortions?’ were only shown to providers, some questions such as: “Did you perform abortions in the past, but not anymore?” were only shown to non-providers.

Attitudes towards abortion were measured via the German version of the validated ‘Abortion Attitude Scale’ [1, 2] containing 14 questions about abortion (e.g. ‘Abortion is wrong no matter what the circumstances are’ or ‘A fetus is not a person until it can live outside its mother’s body’), answered on a 6-point Likert scale from ‘strongly agree’ to ‘strongly disagree’. Seven items were inverted, and a sum score was calculated with a range of 0-70, which was subdivided into five categories (‘strong pro abortion’, ‘moderate pro abortion’, ‘unsure’, ‘moderate contra abortion’, ‘strong contra abortion’).

To measure subjective norms, perceived behavioral control and behavioral intentions (remaining variables of the Theory of Planned Behavior), a manual on constructing items based on TPB [3] was used to develop items on subjective norms of providing abortion (e.g., ‘I feel under social pressure to provide abortions’), perceived behavioral control of providing abortion (e.g., ‘The decision to provide abortions is beyond my control’) and behavioral intentions to provide abortion (e.g., ‘I intend to provide abortions’). These items were answered on a 7-point Likert scale from ‘strongly agree’ to ‘strongly disagree’. The higher the respective value, the higher the subjective norm, the perceived behavioral control and the behavioral intention.

Fear of stigmatization was assessed via the Abortion Provider Stigma Scale (Subscale Disclosure Management) [4] with 10 items such as ‘I (would) avoid telling people what I do for living/ I would be/am afraid of how people will react if they find out about my work in abortion care.’. These items were answered on a 5-point Likert scale from ‘never’ to ‘all of the time’. This scale was adapted to also be used for non-providers: Providers were asked about their actual fear, while non-providers were asked about their anticipated fear.

As a measure of subjective knowledge, participants were asked to rate their knowledge of various aspects of abortion (e.g. ‘I have sufficient theoretical and practical knowledge of abortion to carry it out in practice’ or ‘I consider myself to be well informed about the state laws and regulations on abortion.’) on a 6-point Likert scale from ‘strongly agree’ to ‘strongly disagree’. Two out of six items assessed the general knowledge on abortion, one out of six items assessed the knowledge on current legal regulations of abortions.

### References

1. Lindig A, Christalle E, Zill JM, Baumgart M. Translation, adaptation and psychometric evaluation of the German version of the Abortion Attitude Scale – a secondary analysis of a cross-sectional study among medical students. 2025; doi:10.1101/2025.03.14.25323947

2. Sloan LA. Abortion Attitude Scale. Health education. 1983;14(3):41-2.

3. Francis J. Constructing questionnaires based on the theory of planned behaviour: A manual for health services researchers. 2004. https://openaccess.city.ac.uk/id/eprint/1735/. Accessed 15 Mar 2025

4. Martin LA, Hassinger JA, Seewald M, Harris LH. Evaluation of Abortion Stigma in the Workforce: Development of the Revised Abortion Providers Stigma Scale. Women's health issues : official publication of the Jacobs Institute of Women's Health. 2018;28(1):59-67; doi: 10.1016/j.whi.2017.10.004
