# Supplementary material for: Barriers and motivations: analyzing the factors influencing abortion provision by gynecologists in Germany – a cross-sectional study
Source: Reprod Health. 2026 Jun 18;23:125. doi: 10.1186/s12978-026-02389-8 (PMC13288800; doi:10.1186/s12978-026-02389-8)
Supplement: Supplementary file 4 — Supplementary Material 4. Additional File 4: Survey in its original language. [file 12978_2026_2389_MOESM4_ESM.docx]

## **Fragebogen** zu Einflussfaktoren auf das Angebot von Schwangerschaftsabbrüchen durch niedergelassene Gynäkolog:innen* in Deutschland

*der Fragebogen richtet sich auch an Ärzt*innen, die Schwangerschaftsabbrüche anbieten, aber einer anderen Fachrichtung angehören.

**Im Folgenden möchten wir Ihnen einige Fragen zu Ihrer Person stellen.**

| Wie alt sind Sie? | | | < 30 Jahre | | 30-39 Jahre | | 40-49 Jahre | | | | 50-59 Jahre | ≥ 60 Jahre |  |
| --- | --- | --- | --- | --- | --- | --- | --- | --- | --- | --- | --- | --- | --- |
| Welchem Geschlecht fühlen Sie sich zugehörig? | | | | | | | | |  | | | |  |
|  | weiblich |  | | männlich | | |  | divers/nicht binär | | | | |  |
|  | keine Angabe |  | | anderes *(bitte angeben)* ___________________ | | | | | | | | |  |
| Seit wie vielen Jahren arbeiten Sie als Facharzt/Fachärztin? | | | | | | | ___________________ *(in Jahren)* | | | | | |  |
| In welchem Bundesland arbeiten Sie? | | | | | | ________________________________ | | | | | | |  |
| Wie viele Einwohner:innen hat der Ort, in dem Sie arbeiten? | | | | | | | | | | | | | |
| unter 5.000 Einwohner:innen (Landgemeinde)  5.000 bis unter 20.000 Einwohner:innen (Kleinstadt) | | | | | | 20.000 bis unter 100.000 Einwohner:innen (Mittelstadt)  ab 100.000 Einwohner:innen (Großstadt) | | | | | | | |
| In welchem Bundesland haben Sie in Ihrer Kindheit/Jugend die meiste Zeit gelebt? | | | | | | ________________________________ | | | | | | |  |
| Wie viele Einwohner:innen hat der Ort, in dem Sie in Ihrer Kindheit/Jugend die meiste Zeit gelebt haben? | | | | | | | | | | | | |  |
| unter 5.000 Einwohner:innen (Landgemeinde)  5.000 bis unter 20.000 Einwohner:innen (Kleinstadt) | | | | | | 20.000 bis unter 100.000 Einwohner:innen (Mittelstadt)  ab 100.000 Einwohner:innen (Großstadt) | | | | | | |  |
| Welcher Konfession fühlen Sie sich zugehörig? | | | | | | | | | | | | |  |
| keiner | | | | römisch-katholisch | | | | | | evangelisch | | |  |
| Islam | | | | Judentum | | | | | | Hinduismus | | |  |
| Andere (*bitte angeben*): _____________________ | | | | | | | | | | | | |  |
| Wie stark beeinflussen Ihre religiösen Überzeugungen Ihr Denken und Handeln? | | | | | | | | | | | | |  |
| Ich habe keine religiösen Überzeugungen | | | | | | | | | |  | | |  |
| gar nicht | | | | gering | | | | | | etwas | | |  |
| eher stark | | | | sehr stark | | | | | | Keine Angabe | | |  |
| Wie oft besuchen Sie eine Kirche, einen Tempel, eine Moschee oder andere religiöse Zusammenkünfte? | | | | | | | | | | | | |  |
| nie | | | | einmal im Jahr | | | | | | ein paarmal im Jahr | | |  |
| ein paarmal im Monat | | | | einmal in der Woche oder öfter | | | | | | keine Angabe | | |  |

**Im Folgenden möchten wir gerne erfahren, ob und wie Sie sich in Ihrer Praxis an der Versorgung von Schwangerschaftsabbrüchen beteiligen.**

**Aktuell biete ich […] an:**

| Medikamentöse Schwangerschaftsabbrüche *(mehrere Antworten möglich)* | | | | | |
| --- | --- | --- | --- | --- | --- |
| Ja, Mifepriston/Misoprostol in der Praxis | Ja, im Home use (Misoprostol zu Hause) | Ja, telemedizinisch | | | Nein |
| Wenn JA, seit wann? _________ | | | |  | |
| Operative Schwangerschaftsabbrüche < 12. SSW p.c. *(mehrere Antworten möglich)* | | | | | |
| Ja, mittels Vakuumaspiration | Ja, mittels Kürettage | | | Nein | |
| Wenn JA, seit wann? _________ | | | |  | |
| Operative Schwangerschaftsabbrüche > 12. SSW p.c. | | | | | |
| Ja | Nein | | |  | |
| Wenn JA, seit wann? _________ | | | |  | |
| Schwangerschaftskonfliktberatungen nach §219 StGB mit staatlicher Berechtigung | | | | | |
| Ja | Nein | | |  | |
| Ergebnisoffene Beratungsgespräche für unbeabsichtigt Schwangere | | | | | |
| Ja | Nein | | |  | |
| Haben Sie in der Vergangenheit bereits unbeabsichtigt Schwangere an Ärzt:innen oder eine Praxis/Klinik weitergeleitet, die Schwangerschaftsabbrüche durchführen? | | | | | |
| Ja | Nein | |  |  |  |

**FALLS Sie aktuell KEINE Schwangerschaftsabbrüche in Ihrer Praxis anbieten:**

| Was sind die Hauptgründe, warum Sie keine Schwangerschaftsabbrüche anbieten? *(mehrere Antworten möglich)* | | | | |
| --- | --- | --- | --- | --- |
| Zu geringe Vergütung | ethische/moralische Gründe | religiöse Gründe | | |
| Angst vor Stigmatisierung (negative Bewertung/Verurteilung) | Angst vor Belästigung/Bedrohung | Die Leistung ist zu aufwendig und kostspielig | | |
| Mangelnde praktische Erfahrung | Unsicherheit bei der Durchführung | Personal möchte das nicht | | |
| Unsicherheit durch strafrechtliche Regelung | | | | |
| Andere Gründe (*bitte angeben*) _____________________________________ | | | | |
| Haben Sie in der Vergangenheit Schwangerschaftsabbrüche angeboten? | | | Ja | Nein |
| Falls JA, warum jetzt nicht mehr? *(freiwillige Angabe)* | | | | |
| ________________________________________________________________________________________________________________________________________________________________________________________________________________________________________________________________________ | | | | |

**FALLS Sie aktuell Schwangerschaftsabbrüche in Ihrer Praxis anbieten:**

| Was denken Sie, was sind die Hauptgründe, warum viele Gynäkolog:innen keine Schwangerschaftsabbrüche anbieten? (mehrere Antworten möglich) | | | | |
| --- | --- | --- | --- | --- |
| Zu geringe Vergütung | ethische/moralische Gründe | religiöse Gründe | | |
| Angst vor Stigmatisierung (negative Bewertung/Verurteilung) | Angst vor Belästigung/Bedrohung | Die Leistung ist zu aufwendig und kostspielig | | |
| Mangelnde praktische Erfahrung | Unsicherheit bei der Durchführung | Personal möchte das nicht | | |
| Unsicherheit durch strafrechtliche Regelung | | | | |
| Andere Gründe (*bitte angeben*) _____________________________________ | | | | |
| Sind Sie in der Liste der Bundesärztekammer nach § 13 Abs. 3 Schwangerschaftskonfliktgesetz gelistet? | | | Ja | Nein |
| Falls NEIN, warum nicht? *(freiwillige Angabe)* | | |  |  |
| ________________________________________________________________________________________________________________________________________________________________________________________________________________________________________________________________________ | | | | |

**Am 24. Juni 2022 hat der Bundestag die ersatzlose Streichung des sogenannten Werbeverbots für Schwangerschaftsabbrüche (§219a StGB) beschlossen.**

| Haben Sie **vor** der Abschaffung des §219a StGB auf Ihrer Website über Schwangerschaftsabbrüche informiert? | | |
| --- | --- | --- |
| Ja | Nein | Ich habe keine Website |
| FALLS JA: Worüber haben Sie informiert? | | |
| Methoden des Schwangerschaftsabbruchs | Eigenes Angebot von Schwangerschaftsabbrüchen | Ablauf der Beratung |
| Adressen von Beratungsstellen | Links zu Seiten mit Informationen zum Schwangerschaftsabbruch | Anderes, nämlich ___________ (*bitte angeben*) |
| Haben Sie **nach** der Abschaffung des §219a StGB auf Ihrer Website über Schwangerschaftsabbrüche informiert? | | |
| Ja | Nein | Ich habe keine Website |
| FALLS JA: Worüber haben Sie informiert? | | |
| Methoden des Schwangerschaftsabbruchs | Eigenes Angebot von Schwangerschaftsabbrüchen | Ablauf der Beratung |
| Adressen von Beratungsstellen | Links zu Seiten mit Informationen zum Schwangerschaftsabbruch | Anderes, nämlich ___________ (*bitte angeben*) |

**Im Folgenden geht es um Ihre Einstellungen zum Thema Schwangerschaftsabbruch:**

In den folgenden Aussagen geht es um Ihre Ansichten über **Schwangerschaftsabbrüche während der ersten drei Monate der Schwangerschaft durch eine qualifizierte medizinische Person**. Bitte geben Sie an, wie sehr Sie einer Aussage zustimmen oder sie ablehnen, indem Sie eine Antwortoption ankreuzen. Dies ist kein Test. Es gibt keine richtigen oder falschen Antworten. Bitte antworten Sie so ehrlich wie möglich. **Niemand wird Ihre Antwort ohne Erlaubnis sehen können.**

|  | Stimme voll-kommen zu | Stimme zu | Stimme eher zu | Stimme eher nicht zu | Stimme nicht zu | Stimme über-haupt nicht zu |
| --- | --- | --- | --- | --- | --- | --- |
| In Deutschland sollten Schwangerschaftsabbrüche unter allen Umständen verboten werden. |  |  |  |  |  |  |
| Ein Schwangerschaftsabbruch ist eine gute Möglichkeit, eine ungewollte Schwangerschaft zu beenden. |  |  |  |  |  |  |
| Eine Person sollte sich verpflichtet fühlen, das Kind zu gebären, das gezeugt wurde. |  |  |  |  |  |  |
| Schwangerschaftsabbrüche sind unter allen Umständen falsch. |  |  |  |  |  |  |
| Ein Fötus ist erst eine Person, wenn er außerhalb des Körpers der schwangeren Person leben kann. |  |  |  |  |  |  |
| Die Entscheidung für einen Schwangerschaftsabbruch sollte bei der schwangeren Person liegen. |  |  |  |  |  |  |
| Jedes gezeugte Kind hat das Recht, geboren zu werden. |  |  |  |  |  |  |
| Eine schwangere Person, die kein Kind haben möchte, sollte zu einem Schwangerschaftsabbruch ermutigt werden. |  |  |  |  |  |  |
| Ein Schwangerschaftsabbruch sollte als Tötung eines Menschen gelten. |  |  |  |  |  |  |
| Menschen sollten nicht auf diejenigen herabschauen, die sich für Schwangerschaftsabbrüche entscheiden. |  |  |  |  |  |  |
| Ein Schwangerschaftsabbruch sollte eine leicht zugängliche Alternative für schwangere Minderjährige sein. |  |  |  |  |  |  |
| Niemand sollte die Macht über das Leben oder den Tod eines Fötus haben. |  |  |  |  |  |  |
| Ungewollte Kinder sollten nicht auf die Welt gebracht werden. |  |  |  |  |  |  |
| Ein Fötus sollte ab dem Zeitpunkt der Empfängnis als Person betrachtet werden. |  |  |  |  |  |  |

**Wie stark befürworten oder lehnen Sie ab:**

|  | befürworte stark | befürworte teilweise | weder Ablehnung noch Befürwortung | lehne teilweise ab | lehne stark ab |
| --- | --- | --- | --- | --- | --- |
| … Medikamentöse Schwangerschaftsabbrüche |  |  |  |  |  |
| … Operative Schwangerschaftsabbrüche |  |  |  |  |  |
| … Ein verpflichtendes Beratungsgespräch in einer Beratungsstelle |  |  |  |  |  |
| … Eine verpflichtende Wartezeit zwischen einem Beratungsgespräch und dem Schwangerschaftsabbruch |  |  |  |  |  |
| Die persönliche Wertvorstellung des/der Ärzt/in zum Schwangerschaftsabbruch sollte in die Beratung und Behandlung einfließen. |  |  |  |  |  |
| Ärzt:innen sollten das Recht haben, die Durchführung eines Schwangerschaftsabbruchs zu verweigern |  |  |  |  |  |

**Im Folgenden möchten wir gerne mehr über Ihre Gedanken und Gefühle bezüglich der Durchführung von Schwangerschaftsabbrüchen erfahren.**

| Die meisten Menschen, die mir wichtig sind, denken, dass ich […] Schwangerschaftsabbrüche anbieten sollte. | Auf keinen Fall |  |  |  |  |  |  |  | Auf jeden Fall | |
| --- | --- | --- | --- | --- | --- | --- | --- | --- | --- | --- |
| Für mich ist es […] Schwangerschaftsabbrüche anzubieten. | einfach |  |  |  |  |  |  |  | schwierig | |
| Es wird von mir erwartet, dass ich Schwangerschaftsabbrüche anbiete. | Stimme überhaupt nicht zu |  |  |  |  |  |  |  | Stimme voll zu | |
| Ich empfinde sozialen Druck, Schwangerschaftsabbrüche anzubieten. | Stimme überhaupt nicht zu |  |  |  |  |  |  |  | Stimme voll zu | |
| Ich bin zuversichtlich, dass ich Schwangerschaftsabbrüche anbieten könnte. | Stimme überhaupt nicht zu |  |  |  |  |  |  |  | Stimme voll zu | |
| Die Entscheidung, Schwangerschaftsabbrüche anzubieten, liegt in meiner Hand. | Stimme überhaupt nicht zu |  |  |  |  |  |  |  | Stimme voll zu | |
| Ob ich Schwangerschaftsabbrüche anbiete oder nicht, ist nicht meine alleinige Entscheidung. | Stimme überhaupt nicht zu |  |  |  |  |  |  |  | Stimme voll zu |  |
| Ich gehe davon aus, dass ich (auch) zukünftig Schwangerschaftsabbrüche anbiete. | Stimme überhaupt nicht zu |  |  |  |  |  |  |  | Stimme voll zu |  |
| Ich möchte zukünftig Schwangerschaftsabbrüche anbieten. | Stimme überhaupt nicht zu |  |  |  |  |  |  |  | Stimme voll zu |  |
| Ich beabsichtige, zukünftig Schwangerschaftsabbrüche anzubieten. | Stimme  überhaupt nicht zu |  |  |  |  |  |  |  | Stimme voll zu |  |

Wer oder was hat aus Ihrer Sicht einen Einfluss auf Ihre Entscheidung, Schwangerschaftsabbrüche anzubieten? (*freiwillige Angabe*)

**______________________________________________________________________________________________________________________________________________________________________________________________________________________________________________________________________________**

**Im Folgenden geht es um die wahrgenommene Stigmatisierung im Zusammenhang mit der Durchführung von Schwangerschaftsbrüchen.**

FALLS Sie aktuell Schwangerschaftsabbrüche in Ihrer Praxis durchführen:

**Bitte denken Sie an Ihre Erfahrungen als eine Person, die in der Versorgung von Schwangerschaftsabbrüchen tätig ist.**

Wie häufig finden Sie sich in den folgenden Aussagen wieder?

|  | nie | selten | manchmal | oft | immer |
| --- | --- | --- | --- | --- | --- |
| Die Reaktion anderen Personen darauf, dass ich Schwangerschaftsabbrüche durchführe, bringen mich dazu, mich bedeckt zu halten. |  |  |  |  |  |
| Ich habe das Gefühl, wenn ich anderen Personen von meiner Arbeit erzähle, werden sie mich nur als jemanden betrachten, der:die Schwangerschaftsabbrüche durchführt. |  |  |  |  |  |
| Ich mache mir Sorgen, anderen Personen zu erzählen, dass ich in der Versorgung von Schwangerschaftsabbrüchen arbeite. |  |  |  |  |  |
| Es stört mich, wenn Personen in meiner Nachbarschaft wissen, dass ich in der Versorgung von Schwangerschaftsabbrüchen arbeite. |  |  |  |  |  |
| Ich vermeide es, den Menschen zu erzählen, womit ich meinen Lebensunterhalt verdiene. |  |  |  |  |  |
| Ich habe Angst, dass ich mich selbst oder meine Angehörigen Gewaltgefahren aussetze, wenn ich erzähle, dass ich in der Versorgung von Schwangerschaftsabbrüchen tätig bin. |  |  |  |  |  |
| Ich habe das Gefühl, dass eine Offenlegung meiner Arbeit den möglichen Ärger nicht wert ist, der sich daraus ergeben könnte. |  |  |  |  |  |
| Ich habe Angst, wie Personen reagieren, wenn sie herausfinden, dass ich in der Versorgung von Schwangerschaftsabbrüchen tätig bin. |  |  |  |  |  |
| Ich habe das Gefühl, dass ich meine Arbeit in der Versorgung von Schwangerschaftsabbrüchen vor meinen Freund:innen verheimlichen muss. |  |  |  |  |  |
| Es fällt mir schwer, anderen davon zu erzählen, dass ich in der Versorgung von Schwangerschaftsabbrüchen tätig bin. |  |  |  |  |  |
| Ich habe Angst vor Kriminalisierung durch die aktuelle rechtliche Regelung des Schwangerschaftsabbruchs |  |  |  |  |  |

FALLS Sie aktuell KEINE Schwangerschaftsabbrüche in Ihrer Praxis durchführen:

**Bitte stellen Sie sich vor, welche Erfahrungen Sie machen würden, wenn Sie in der Versorgung von Schwangerschaftsabbrüchen tätig wären.**Wie häufig würden Sie sich in den folgenden Aussagen wiederfinden?

|  | nie | selten | manchmal | oft | immer |
| --- | --- | --- | --- | --- | --- |
| Die Reaktion anderer Personen darauf, dass ich Schwangerschaftsabbrüche durchführe, würden mich dazu bringen, mich bedeckt zu halten. |  |  |  |  |  |
| Ich hätte das Gefühl, wenn ich anderen Personen von meiner Arbeit erzähle, werden sie mich nur als jemanden betrachten, der:die Schwangerschaftsabbrüche durchführt. |  |  |  |  |  |
| Ich würde mir Sorgen machen, anderen Personen zu erzählen, dass ich Schwangerschaftsabbrüche durchführe. |  |  |  |  |  |
| Es würde mich stören, wenn Personen in meiner Nachbarschaft wüssten, dass ich Schwangerschaftsabbrüche durchführe. |  |  |  |  |  |
| Ich würde es vermeiden, Personen zu erzählen, dass ich meinen Lebensunterhalt unter anderem mit der Durchführung von Schwangerschaftsabbrüchen verdiene. |  |  |  |  |  |
| Ich hätte Angst, dass ich mich selbst oder meine Angehörigen Anfeindungen aussetze, wenn ich erzählte, dass ich Schwangerschaftsabbrüche durchführe. |  |  |  |  |  |
| Ich hätte das Gefühl, dass eine Offenlegung meiner Arbeit den möglichen Ärger nicht wert wäre, der sich daraus ergeben könnte. |  |  |  |  |  |
| Ich hätte Angst, wie Personen reagieren, wenn sie herausfinden, dass ich Schwangerschaftsabbrüche durchführe. |  |  |  |  |  |
| Ich hätte das Gefühl, dass ich meinen Freund:innen verheimlichen muss, dass ich Schwangerschaftsabbrüche durchführe. |  |  |  |  |  |
| Es würde mir schwer fallen, anderen davon zu erzählen, dass ich Schwangerschaftsabbrüche durchführe. |  |  |  |  |  |
| Ich habe / hätte Angst vor Kriminalisierung durch die aktuelle rechtliche Regelung des Schwangerschaftsabbruchs |  |  |  |  |  |

**Im Folgenden geht es darum, wie Sie Ihr Wissen zum Schwangerschaftsabbruch einschätzen.**

Bitte geben Sie an, inwieweit Sie jeder der folgenden Aussagen zustimmen oder ablehnen.

|  | | Stimme voll-kommen zu | | Stimme zu | | Stimme eher zu | | Stimme eher nicht zu | | Stimme nicht zu | Stimme überhaupt nicht zu | |
| --- | --- | --- | --- | --- | --- | --- | --- | --- | --- | --- | --- | --- |
| Ich habe ausreichendes theoretisches Wissen zum Schwangerschaftsabbruch, um Patient:innen zu diesem Thema zu beraten. | |  | |  | |  | |  | |  |  | |
| Ich habe ausreichendes theoretisches und praktisches Wissen zum Schwangerschaftsabbruch, um diese in der Praxis durchzuführen. | |  | |  | |  | |  | |  |  | |
| Ich kenne die Inhalte der S2k-Leitlinie Schwangerschaftsabbruch im 1. Trimenon (2023). | |  | |  | |  | |  | |  |  | |
| Ich weiß, welche Methoden des Schwangerschaftsabbruchs von der WHO (World Health Organisation) empfohlen werden. | |  | |  | |  | |  | |  |  | |
| Ich weiß, wo ich erlernen kann, Schwangerschaftsabbrüche durchzuführen. | |  | |  | |  | |  | |  |  | |
| Ich betrachte mich als gut informiert über die staatlichen Gesetze und Regelungen zum Schwangerschaftsabbruch. | |  | |  | |  | |  | |  | |  |

**Im Folgenden geht es um Ihre Ausbildung zu Methoden des Schwangerschaftsabbruchs.**

| Hätten Sie sich gewünscht, im Studium mehr über die Durchführung von Schwangerschaftsabbrüchen zu lernen? | Ja | Nein |
| --- | --- | --- |
| Hätten Sie sich gewünscht, in der Facharztausbildung mehr über die Durchführung von Schwangerschaftsabbrüchen zu lernen? | Ja | Nein |
| Welche Methoden des **induzierten Schwangerschaftsabbruchs** haben Sie in Ihrer Facharztausbildung gelernt? | | |
| Durchführung des medikamentösen Schwangerschaftsabbruchs | | |
| Durchführung einer Vakuumaspiration für einen induzierten Schwangerschaftsabbruch | | |
| Durchführung einer Kürettage für einen induzierten Schwangerschaftsabbruch | | |

**Im Folgenden geht es um Ihre Einschätzung der Versorgungslage in Deutschland in Bezug zum Schwangerschaftsabbruch.**

Bitte geben Sie an, inwieweit Sie jeder der folgenden Aussagen zustimmen oder ablehnen.

|  | Stimme voll-kommen zu | Stimme zu | Stimme eher zu | Stimme eher nicht zu | Stimme nicht zu | Stimme über-haupt nicht zu |
| --- | --- | --- | --- | --- | --- | --- |
| Es gibt in Deutschland ausreichend Einrichtungen, die Schwangerschaftsabbrüche vornehmen. |  |  |  |  |  |  |
| Unbeabsichtigt Schwangere haben in Deutschland leichten Zugang zu Schwangerschaftsabbrüchen. |  |  |  |  |  |  |
| Unbeabsichtigt Schwangere haben in Deutschland einen leichten Zugang zu Informationen zu Schwangerschaftsabbrüchen |  |  |  |  |  |  |
| Der Zugang zu Verhütungsmitteln in Deutschland ist leicht |  |  |  |  |  |  |

**Abschließend geht es um Ihr Wissen zu den aktuellen gesetzlichen Regelungen zum Schwangerschaftsabbruch und zu Folgen von Schwangerschaftsabbrüchen.**

Bitte beachten Sie, dass der folgende Abschnitt kein Test ist. Informationen über Ihr Wissen zu gesetzlichen Regelungen zu erhalten, hilft uns, Einflussfaktoren auf die aktuelle Versorgungslage von unbeabsichtigten Schwangerschaften in Deutschland besser zu verstehen. Ihre ehrlichen Antworten sind für uns äußerst wertvoll.

Bitte geben Sie mit Hilfe der Antwortoptionen an, ob die Aussagen Ihrer Meinung nach richtig oder falsch sind.

Die Aussagen beziehen sich auf Schwangerschaftsabbrüche, die in Deutschland auf Grund der Beratungsregelung durchgeführt werden.

|  | Ja | Nein | Ich weiß es nicht |
| --- | --- | --- | --- |
| Institutionen dürfen die Durchführung verweigern. |  |  |  |
| Jede:r Ärzt:in darf die Durchführung verweigern. |  |  |  |
| Der Arzt/die Ärztin, der/die den Abbruch der Schwangerschaft vornimmt, kann auch die Schwangerschaftskonfliktberatung durchführen. |  |  |  |
| Nach dem Beratungsgespräch kann die Schwangere am nächsten Tag den Abbruch durchführen lassen. |  |  |  |
| Die Kosten für den Schwangerschaftsabbruch (nach Beratungsregelung) übernimmt grundsätzlich die Krankenkasse. |  |  |  |
| Schwangerschaftsabbrüche sind mit einem erhöhten Risiko für Brustkrebs verbunden. |  |  |  |
| Schwangerschaftsabbrüche haben fast immer negative psychologische Auswirkungen. |  |  |  |
| Schwangerschaftsabbrüche erschweren es, in Zukunft schwanger zu werden. |  |  |  |
| Schwangerschaftsabbrüche erhöhen das Risiko für zukünftige Fehlgeburten |  |  |  |

| Ich habe alle Fragen ernsthaft und nach bestem Gewissen beantwortet. | Ja  Nein |
| --- | --- |

## Vielen Dank für das Ausfüllen des Fragebogens!

Herzlichen Dank für Ihre Teilnahme und Ihren Beitrag zur Studie! Mehr Informationen zur CarePreg Studie erhalten Sie unter [www.uke.de/carepreg](http://www.uke.de/carepreg)

Sollten Sie noch Fragen zur Studie haben, wenden Sie sich gerne an Vanessa Le (v.le@uke.de) oder Dr. Anja Lindig (a.lindig@uke.de, Tel.: 040-7410-59893).

**Interesse an unserem Newsletter?**

Wenn Sie weiter über den Verlauf unserer Studie informiert werden möchten, schreiben Sie uns über **carepreg@uke.de**, um in unseren Newsletter-Verteiler aufgenommen zu werden.

**Das CarePreg Team**

Vanessa Le (*Projektmitarbeiterin*)

Dr. Anja Lindig (*Projektleitung*)
